# Pilomatrixoma of the Arm: A Rare Case with Cytologic Diagnosis

**DOI:** 10.1155/2012/257405

**Published:** 2012-10-04

**Authors:** Ruchika Gupta, Sarika Verma, Pankaj Bansal, Anup Mohta

**Affiliations:** ^1^Department of Pathology, Chacha Nehru Bal Chikitsalaya, Maulana Azad Medical College, Geeta Colony, Delhi 110031, India; ^2^Department of Pediatric Surgery, Chacha Nehru Bal Chikitsalaya, Maulana Azad Medical College, Geeta Colony, Delhi 110031, India

## Abstract

Pilomatrixoma, a benign skin appendageal tumor, is seen commonly in head and neck. Occurrence of pilomatrixoma in the upper extremities is not common and has been reported infrequently in the available literature. Only a few cases with preoperative aspiration cytology have been reported in the literature. A five-year-old girl underwent fine needle aspiration (FNA) of a firm subcutaneous nodule on the lateral aspect of left arm. FNA smears showed scattered and few fragments of round to oval cells along with multinucleated giant cells. Few shadow cells were seen. A cytologic impression of pilomatrixoma was rendered, which was confirmed on histopathology. Pilomatrixoma, a common skin appendageal tumor in head and neck region, should be considered in the cytologic differential diagnoses of subcutaneous masses even in unusual locations like arm. The varied cytomorphology should be remembered to avoid misdiagnosis.

## 1. Introduction

Pilomatrixoma, also known as calcifying epithelioma of Malherbe, is a skin appendageal tumor seen in head and neck regions. Occurrence of this lesion in the arm is unusual and has been described in few reports in the available English literature [1–4]. Though histologic diagnosis of pilomatrixoma, even in unusual locations, is straightforward, the same is not true for aspiration cytology. There have been quite a few reports of misdiagnosis of pilomatrixoma on aspiration smears, as other benign as well malignant lesions [5, 6]. One case of pilomatrixoma of the arm was diagnosed as round cell tumor on cytology. The final diagnosis was rendered on histopathology of the resected mass [3]. An accurate diagnosis of this benign lesion on cytology is imperative, considering that excision is curative. 

We describe the clinical, cytologic, and histologic features of a case of pilomatrixoma in the arm of a young girl. 

## 2. Case Report

A five-year-old girl presented to the pediatric surgery outpatient department with history of gradually increasing swelling in the left arm for the last 3-4 months. There was no associated pain or history of trauma prior to the appearance of the swelling. Local examination showed a firm subcutaneous swelling, 0.8 × 0.6 cm in size on the lateral aspect of the left arm. The swelling was nontender with no fixity to the overlying skin or underlying structures. The overlying skin appeared unremarkable. With a clinical diagnosis of a soft tissue lesion, fine needle aspiration (FNA) was performed from the swelling. Following the cytologic impression, an excision of the mass was carried out. 

### 2.1. Cytologic Features

**Figure 1:**
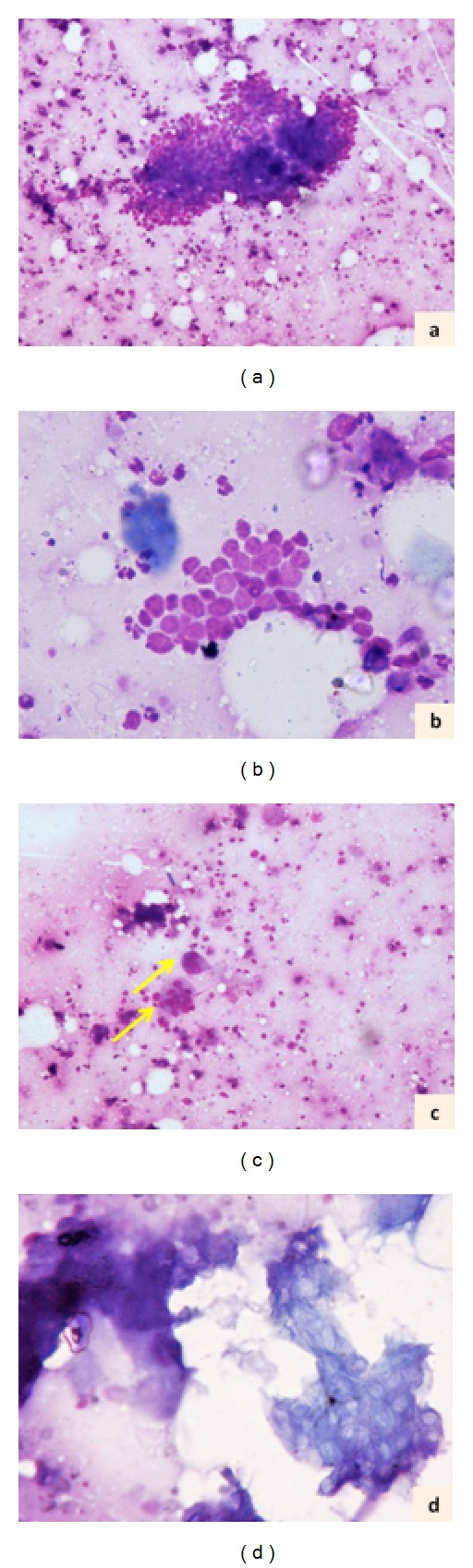
Panel of cytologic photomicrographs showing fragment of round to oval cells ((a), Giemsa ×100), better seen at higher magnification ((b), Giemsa ×200). Multinucleated giant cells (arrow) with background showing scattered oval cells ((c), Giemsa ×100). An area showing “shadow” cells ((d), Giemsa ×200).

FNA was performed using 22 G needle and 10 mL syringe. The smears were air dried and stained with Giemsa stain. FNA smears showed singly-lying as well as few aggregates of round to oval cells with scant to moderate amount of pale blue cytoplasm, vesicular nuclei with occasional cell showing prominent nucleolus (Figures 1(a) and 1(b)). Admixed multinucleated giant cells were also noted (Figure 1(c)). Few shadow cells were also noted (Figure 1(d)). Mitotic activity or necrosis was not identified in the smears examined. A cytologic diagnosis of pilomatrixoma was rendered and excision biopsy advised. 

### 2.2. Histopathologic Features

**Figure 2:**
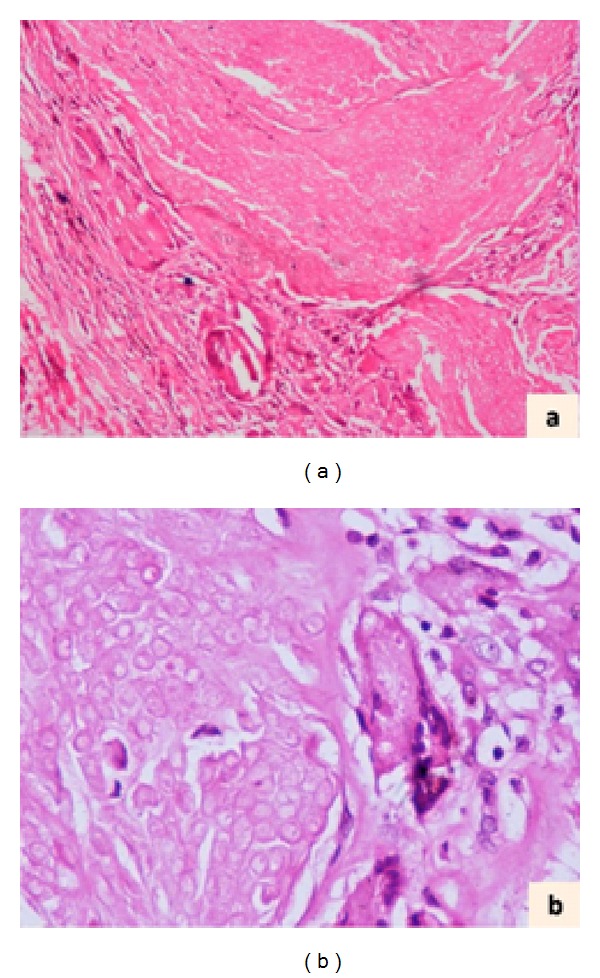
Histologic photomicrographs showing islands of shadow cells surrounded by multinucleated giant cells ((a), H&E ×100). Higher magnification demonstrates shadow cells better ((b), H&E ×200).

We received a nodular soft tissue measuring 0.6 × 0.5 × 0.3 cm with attached skin 0.5 × 0.3 cm. Cut section showed a circumscribed grey-white lesion 0.4 cm in diameter. Sections from the lesion showed features of pilomatrixoma with shadow cells, basaloid cells, and multinucleated giant cells (Figures 2(a) and 2(b)). 

## 3. Discussion

Pilomatrixoma (calcifying epithelioma of Malherbe) is a benign skin appendageal tumor with differentiation towards hair follicle matrix cells. This lesion occurs over a wide age range with two peaks: less than 20 years and over 50 years [7]. Pilomatrixoma is typically found in head and neck region, though it has been reported in upper extremities and other sites. In a large series of 346 pilomatrixomas, about 15.3% were seen in upper extremities [8]. There have been few reports of pilomatrixoma occurring in the arm in the existing literature [1–4]. Clinically, pilomatrixoma presents as solitary painless and well circumscribed dermal or subcutaneous mass upto 3 cm in diameter [9]. The overlying skin may display telangiectasia, hemangioma-like color, or blue-black discoloration [10]. Our patient, a five-year girl, had a solitary firm lesion on the lateral aspect of left arm with no changes in the overlying skin. 

Fine needle aspiration cytology (FNAC), the most favoured diagnostic modality in superficial masses, usually shows characteristic features of pilomatrixoma. These include basaloid cell clusters, shadow (ghost) cells, calcification, and few nucleated squamous cells. Giant cells may be seen in response to keratin [5]. Despite these features, pilomatrixoma may be mistaken for other skin lesions [6]. A previously reported case of pilomatrixoma of the arm was diagnosed cytologically as blue round cell tumor due to the presence of round to ovoid cells with occasional rosette-like appearance. Histopathology in this case showed features of pilomatrixoma [3]. The authors suggested that early rapidly growing lesions, composed predominantly of basaloid cells, may lead to overdiagnosis of malignancy [3]. 

Histologic features of pilomatrixoma are well described in the literature as a deep subepidermal tumor consisting of irregular islands of keratinized shadow cells surrounded by peripheral basaloid cells. Calcification may be seen in shadow cell regions along with foreign body giant cell reaction to keratin [9]. 

## 4. Conclusion

The present case highlights the importance of considering pilomatrixoma in the clinical and pathologic differential diagnosis of dermal or subcutaneous nodule even in locations other than head and neck region. This is true especially for cytopathologists since they play an important role in the initial diagnosis of these lesions. Cytopathologists should keep in mind the variability of the cytologic features, given the cellular composition of the lesion.
